# Effects of Light Quality on Colonization of Tomato Roots by AMF and Implications for Growth and Defense

**DOI:** 10.3390/plants11070861

**Published:** 2022-03-24

**Authors:** Haymanti Saha, Nikolaos Kaloterakis, Jeffrey A. Harvey, Wim H. Van der Putten, Arjen Biere

**Affiliations:** 1 Department of Terrestrial Ecology, Netherlands Institute of Ecology (NIOO-KNAW), Droevendaalsesteeg 10, 6708 PB Wageningen, The Netherlands; n.kaloterakis@fz-juelich.de (N.K.); j.harvey@nioo.knaw.nl (J.A.H.); w.vanderputten@nioo.knaw.nl (W.H.V.d.P.); a.biere@nioo.knaw.nl (A.B.); 2 Soil Biology Group, Wageningen University and Research, Droevendaalsesteeg 2, 6708 PB Wageningen, The Netherlands; 3 Institute of Bio- and Geosciences, Agrosphere (IBG-3), Forschungszentrum Jülich GmbH, Wilhelm-Johnen-Straße, 52428 Jülich, Germany; 4 Department of Ecological Sciences, Section Animal Ecology, Vrije Universiteit Amsterdam, De Boelelaan 1085, 1081 HV Amsterdam, The Netherlands; 5 Laboratory of Nematology, Wageningen University and Research, Droevendaalsesteeg 2, 6708 PB Wageningen, The Netherlands

**Keywords:** arbuscular mycorrhizal fungi, context dependency, light quality, plant defense, plant microbe insect interactions, *Solanum lycopersicum* (tomato)

## Abstract

Beneficial soil microbes can enhance plant growth and defense, but the extent to which this occurs depends on the availability of resources, such as water and nutrients. However, relatively little is known about the role of light quality, which is altered during shading, resulting a low red: far-red ratio (R:FR) of light. We examined how low R:FR light influences arbuscular mycorrhizal fungus (AMF)-mediated changes in plant growth and defense using *Solanum lycopersicum* (tomato) and the insect herbivore *Chrysodeixis chalcites*. We also examined effects on third trophic level interactions with the parasitoid *Cotesia marginiventris*. Under low R:FR light, non-mycorrhizal plants activated the shade avoidance syndrome (SAS), resulting in enhanced biomass production. However, mycorrhizal inoculation decreased stem elongation in shaded plants, thus counteracting the plant’s SAS response to shading. Unexpectedly, activation of SAS under low R:FR light did not increase plant susceptibility to the herbivore in either non-mycorrhizal or mycorrhizal plants. AMF did not significantly affect survival or growth of caterpillars and parasitoids but suppressed herbivore-induced expression of jasmonic acid-signaled defenses genes under low R:FR light. These results highlight the context-dependency of AMF effects on plant growth and defense and the potentially adverse effects of AMF under shading.

## 1. Introduction

In natural ecosystems, ecological communities are characterized by multitrophic interactions, e.g., between plants and their above- and belowground beneficial insects and microbes, pests and pathogens [1,2]. An important groups of beneficial plant root symbionts are Arbuscular Mycorrhizal Fungi (AMF). The beneficial potential of AMF for plant growth and fitness has received considerable attention over the past decades. AMF form hyphal networks with plant roots that enhance the access of roots to a large soil surface area, often resulting in enhanced plant growth and a positive mycorrhizal growth response (MGR) [3]. In addition to providing nutritional benefits, AMF colonization is also known to enhance tolerance to abiotic stresses such as drought and salinity and to improve direct and indirect plant defenses against pathogens and pests [4,5,6,7,8]. One of the mechanisms underlying enhanced biotic stress resistance is Mycorrhiza-Induced Resistance (MIR) that is based on Induced Systemic Resistance (ISR), a form of defense priming [9] that sensitizes the plant’s immune system, leading to enhanced plant direct and indirect defense [10].

The use of MIR to reduce pest damage in agriculture has emerged as a highly promising avenue to improve crop resilience and reduce pesticide use [11]. Several studies have demonstrated that mycorrhizal fungi can enhance plant tolerance and resistance to above-ground herbivory [10,12,13] and that this can be mediated by priming of jasmonic acid (JA)-signaled defense responses [14]. Mycorrhizal fungi generally decrease the performance of non-specialist chewing insects that feed on a variety of plant species [9]. In contrast, specialist insects usually perform better on mycorrhizal plants, probably because specialists have evolved unique detoxifying enzymes that counteract the effects of plant secondary metabolites [15,16] and can benefit from an increased nutritional status of mycorrhizal plants. However, the outcome of plant-microbe-insect interactions is often association-specific, and depends on the identities of the beneficial microbes, plant hosts, insect species and their stages of development involved [17,18,19].

The positive effect of AMF on plant growth that is often observed has stimulated the development of a wide range of biostimulant products for application in agriculture [20,21]. However, it is now recognized that interactions between plants and AMF are not always beneficial to the plant and that the effects of AMF on plants range from mutualism (positive) to neutral or parasitism (negative) [22], depending on species or strain combinations and abiotic or biotic environmental conditions. This context-dependency of AMF has been shown both for AMF-induced growth responses and for AMF-induced defense responses [20,23,24], highlighting the unpredictability of the functional responses of AMF in terms of induced resistance.

Although our knowledge of the environmental factors that predominantly influence the context-dependency of MIR is still unresolved, recent studies have identified a range of abiotic and biotic factors that affect the successful triggering of ISR by beneficial microbes such as of AMF against particular pest species. Biotic factors include for instance the identity of the insect pest in response to which the microbially-induced response should be triggered [17,25,26]. Furthermore, abiotic stresses can modify the effects of mycorrhizal fungi on plant growth and defense in at least two ways. First, for a beneficial microbe to improve plant tolerance to a stress, the plant–microbe symbiosis needs to be established under those stress conditions [27]. Low light availability [28] or quality [29], drought [30], high nutrient availability [31], or high temperature [32] may decrease colonization by beneficial microbes, although this does not necessarily impair the resistance-inducing effects of the microbes [33]. In contrast, low nutrient levels, especially phosphorus, often enhance colonization by AMF, increasing the opportunities for triggering ISR. Second, for any given level of host colonization, abiotic conditions may affect the functionality of the mycorrhizal association in terms of activation of ISR, e.g., through changes in host physiology that may affect the response to ISR-triggering microbes [30,34,35].

Plants are often shaded by neighbors, causing changes in both the quantity of intercepted light and in light quality, which can have important consequences for plant-microbe interactions [28]. Recent studies have shown that plant responses to shading have important consequences for growth-defense trade-offs [36]. Shading lowers the ratio of red to far-red (R:FR) light that is perceived by plant phytochrome B (PhyB) receptors [37]. Plants perceiving a low R:FR light ratio due to shading-induced inactivation of PhyB activate a so-called Shade Avoidance Syndrome (SAS) [38], resulting in elongation growth in order to improve competition with neighbors for available light. Recent studies have shown that activation of SAS can impair jasmonic acid (JA) signaled defenses due to negative interactions between repressor proteins of gibberellic acid signaling (DELLAs) involved in SAS and repressor proteins of jasmonic acid signaling (JAZs) involved in defense [39,40,41]. Consequently, plants prioritizing the activation of SAS under shade often show a reduced ability to mount defenses against pathogens and herbivores that are signaled through the JA defense pathway [36,42,43].

Light conditions also strongly modulate the outcome of AMF symbiosis for host plant growth [28]. Both light quality and quantity can affect colonization by mycorrhizal fungi. Notably, light deprivation leads to a reduction in root colonization by AMF [28] and this can modify the effect of AMF on its host plant, ranging from positive to neutral or negative [44]. Furthermore, light quality (low R:FR) has been shown to reduce ectomycorrhization and root growth of Scots pine seedlings [45]. However, we lack an understanding of whether and how light quantity and quality affect the impact of the mycorrhizal symbiosis on plant defense.

This is not only true for effects on the resistance to herbivorous insects (direct defense), but also for effects on the natural enemies of these herbivores (indirect defense). Evidence suggests that AMF can impact plant indirect defense through cascading effects of AMF colonization on higher trophic levels. For instance, AMF can affect both the rate of aphid parasitism by a parasitic wasp and parasitoid preference [46], likely mediated by AMF-induced changes in the production of herbivore-induced plant volatiles [47]. Furthermore, the performance of parasitoid wasps often varies with the quality of its host’s diet [48], and the development of aphid parasitoids was shown to depend on the presence and identity of AMF [5]. However, we lack insight in the effects of abiotic factors on the impact of beneficial microbes on insect pest natural enemies.

In the present study we examine the effects of ambient vs. low R:FR light on the AMF colonization of tomato plants and the interactive effects of light quality and AMF on plant growth and the performance of an insect herbivore and one of its natural enemies. The latter is studied by assessing effects on a parasitoid wasp developing inside its host herbivore (i.e., an endoparasitoid [49]). Specifically, we address four questions: (1) Does low R:FR light alter mycorrhizal colonization in tomato plants? (2) What is the effect of light quality, AMF and herbivory on plant growth and leaf nutrient content? (3) Does AMF induce defense against the insect pest, and does it mitigate the growth-defense trade off during SAS? (4) Is the impact of light and AMF on the herbivore reflected in effects on the third trophic level? We hypothesize that (i) low R:FR light will reduce mycorrhizal colonization; (ii) AMF will improve nutrient uptake whereas light quality and herbivory will interactively influence plant nutrient allocation; (iii) low R:FR light will activate SAS, resulting in decreased plant resistance to herbivory especially in non-mycorrhizal plants, whereas AMF will induce MIR under both ambient and low R:FR conditions (iv) AMF will positively affect parasitoid development under both light conditions.

## 2. Materials and Methods

### 2.1. Plants and Soil

*Solanum lycopersicum* cultivar Moneymaker (Buzzy Seeds, Volendam, The Netherlands) seeds were surface sterilized with 1% sodium hypochlorite for 10 min, followed by 70% ethanol for 3 min and rinsed five times with sterile demineralized water. Air dried seeds were sown in autoclaved sand-soil mixture (4:1, v:v) in 3.7 cm × 4.0 cm (hole diameter × depth) cell seedling trays and placed under the light treatments. Low nutrient natural soil was obtained from an abandoned field in Dennenkamp (52°01′31.6° N 5°48′24.6° E), the Netherlands (mineral P-CaCl2 = 0.3 ppm, mineral N-CaCl2 = 8.1 ppm). Middle coarse sand (0.71–1.25 mm) (Wildkamp, Lutten, The Netherlands), was oven-sterilized prior to the experiment.

### 2.2. Fungi and Insects

The arbuscular mycorrhizal fungus *Rhizoglomus irregularis* MUCL-57021, was acquired from Koppert B.V. (Berkel en Rodenrijs, The Netherlands) as spore suspension in sterilized water. Eggs of the tomato looper *Chrysodeixis chalcites* (*Lepidoptera: Noctuidae*), a notorious noctuid pest found in Dutch tomato glasshouse, were obtained from Wageningen University and Research Center in Bleiswijk, The Netherlands. The caterpillars were reared on tomato leaves at 22–24 °C and 16 h photoperiod and 50% relative humidity. Pupae were transferred to plexiglass cylinders with vermiculite, lined with filter paper. This created an artificial environment for the moths to lay eggs. Adult moths were reared on honey water solution.

Cocoons of *Cotesia marginiventris* (Hymenoptera: Braconidae), a solitary, koinobiont, endoparasitoid, were obtained from the University of Neuchâtel, Switzerland, where they had been reared on the host *Spodoptera exigua* (Lepidoptera: Noctuidae). Parasitoid cocoons were incubated in a climate cabinet at 22 °C until emergence. Adult parasitoids were kept at 10 °C with 16 h photoperiod and 50% humidity inside insect cages (15 cm × 15 cm × 15 cm) with honey water.

Parasitized caterpillars were prepared to study the development of parasitoids on host caterpillars feeding on plants from the different light and mycorrhiza treatments. One randomly selected female *C. marginiventris* parasitoid and one second instar *C. chalcites* larva were placed together in a plastic vial until oviposition was observed. 144 caterpillars were parasitized and maximum 3 caterpillars were parasitized by the same female parasitoid. Individual unparasitized and parasitized caterpillars were placed in plastic vials and starved overnight before introducing them on the leaves of the experimental plants.

### 2.3. Experimental Setup

The experiment was carried out in the late spring of 2019 in the glasshouse facility at the Netherlands Institute of Ecology, Wageningen, The Netherlands. The experiment followed a factorial design with two light treatments (Ambient; Low R:FR), two mycorrhizal treatments (AMF−: mock inoculation; AMF+: *R. irregularis* spores), three herbivory treatments (C: no herbivory; H: herbivory by unparasitized caterpillars; HP: herbivory by parasitized caterpillars) and 18 replicates per treatment combination, totaling 216 plants.

The experiment was arranged in a split plot design with light as whole-plot factor. Six pairs of carts (blocks) were set up. Each cart (70 × 100 cm) contained 18 plants (three biological replicates from each mycorrhiza/herbivory combination). One cart from each block was assigned to the ambient light treatment, the other to the low R:FR light treatment. However, during the herbivory experiment one block had to be discarded due to unexpected browning of some of the plants.

Light treatments were created by mounting a rectangular wooden frame (100 × 70 cm) on top of each cart at a height of 70 cm, that either contained no LED lights (ambient light condition), or 30 equidistant and inward directed FR-emitting LED lights (low R:FR light condition; peak 730 nm, 15 W/m^2^ in total, Philips GreenPower; Koninklijke Philips N.V., Amsterdam, The Netherlands). The LEDs were switched on between 05:30 h and 22: 30 h and reduced the R:FR ratio on average from c. 1.0 to c. 0.3, varying with external light conditions (Figure S1). 250 tomato seeds were sown individually in seedling trays as described above and were either inoculated with one ml of a freshly prepared suspension containing 1000 *R. irregularis* spores (AMF+ treatments) or ‘mock’ inoculated (one ml of autoclaved demineralized water; Myc− treatments).

### 2.4. Growth Conditions

Two weeks after sowing, 108 uniform seedlings from both the AMF+ and the AMF− seedling trays were selected and transplanted with their seedling soil into 1 litre pots (10 × 10 × 11 cm) placed on the carts. The pots were filled with 750 mL of the sterilized sand-soil mixture described above, supplemented with 0.5 g/0.01 m2 organic phosphate fertilizer supplied as bone meal (16% P_2_O_5_, Ecostyle, Oosterwolde, The Netherlands). Glasshouse conditions were set to 16 h: 8 h photoperiod (light: dark), constant temperature (22 ± 2 °C) and 70% relative humidity. Additional lighting was provided between 06:00 h and 22:00 h by high pressure Sodium lamps (Son-T, 600 W Philips GreenPower; Koninklijke Philips N.V., Amsterdam, The Netherlands) that were automatically switched on when light intensity dropped below 225 µmol m^−2^ s^−1^. Carts were rotated every week to account for spatial variation in temperature and humidity inside the glasshouse compartment. Plants were irrigated every other day with demineralized water. Half strength Hoagland nutrient solution without potassium phosphate (KH_2_PO_4_) was supplemented, initially once a week, and then twice a week in later plant growth stages. As mentioned above, phosphate was supplied in the form of bone meal. Plants generally have more difficulty accessing organic phosphate than mineral phosphate, the form in which it is supplied in Hoagland solution. Since mycorrhizae can help in solubilizing and taking up phosphate from organic sources, supplying P as bone meal is expected to enhance the association of the plant with its mycorrhizal symbiont and fungal colonization [50].

### 2.5. Insect Bioassay

Three weeks after seedling transplantation, bioassays were initiated using parasitized and unparasitized second instar larvae of *C. chalcites* to assess effects on herbivore and natural enemy development on plants from the different mycorrhiza and light treatments. Newly hatched caterpillars were initially reared on detached leaves of extra plants from each of the four mycorrhiza and light treatment combinations in growth chambers (24 °C, 16 h photoperiod, 22 °C at night, RH = 60%). Freshly molted second instar caterpillars were divided into parasitized (by *C. marginiventris*) and unparasitized. All caterpillars were put into individual vials and starved overnight.

The following day caterpillars were weighed on a micro balance and individually placed in a clip cage (⌀ 8 cm) on the second true leaf from the bottom, enclosing one tomato leaflet. Control plants received empty cages. The individual fresh weight of the caterpillar was recorded every third day during the 2-week bioassay.

For the parasitized herbivores, the parasitoid cocoons were collected by day, weighed, and observed until wasp emergence. We recorded wasp development time (time between parasitation and cocoon production), eclosion period, weight of adult wasps, and their sex. After each caterpillar weight measurement, the surviving larvae were placed on a new leaflet on the same leaf. When the whole leaf was consumed all the caterpillars were placed on a new, one younger leaf. Two weeks post feeding on the plants, caterpillars were removed, and final fresh weight was recorded. Caterpillars were frozen and subsequently dried in an oven at 60 °C until constant weight and their dry weight was recorded.

### 2.6. Harvest Measurements

Following transplantation, plant development was monitored for five weeks by measuring plant height. At harvest, eight weeks after seed sowing and inoculation, all aboveground plant material was harvested on a single day. Roots were harvested and cleaned over a period of three days, while being stored at 4 °C to arrest rapid dying of roots. For leaf gene expression analyses, one systemic leaf (one younger than the leaves exposed to herbivory) was selected from herbivore-exposed plants (H), and a corresponding ontogenetic leaf was selected from plants in the non-herbivore treatment (C). From each of these leaves, six 8 mm diameter leaf discs were collected using a sterilized cork borer and immediately flash frozen. Sampled leaves from plants exposed to parasitized herbivores (HP) were not used for analysis. Three individual biological replicates were pooled into a single sample for each treatment combination. A total of five pooled replicates of 18 leaf discs were sampled per treatment.

Fresh weight (FW) of leaves, stems, inflorescences, and washed roots was measured separately for every individual plant. Total FW of roots was recorded after dabbing them between two layers of tissue paper, before selecting and cutting lateral fine roots from three different sections (top, middle, bottom region) of c. 1.5 cm for counting mycorrhizal colonization. Cut sections were then placed in 15 mL tubes with 70% ethanol and stored at 4 °C until staining. FW of the leftover roots were recorded to allow estimation of the total root dry weight based on the product of the total root FW and the DW:FW ratio of the leftover roots. All AG and BG plant parts were oven dried for 72 h at 60 °C to estimate dry weight of individual plants.

### 2.7. Root Staining and Microscopy

To check mycorrhizal colonization, root segments were cut into 1.0 cm pieces, cleaned in 10% 10 KOH for 10 min at 90 °C, rinsed in water, acidified with 1% HCl and stained with acidified ink stain (Royal Blue, Parker ink) for 15 min at 90 °C [51]. After de-staining overnight, 30 random uniform root sections were taken, and individual root segments were arranged on glass microscopy slides (three slides with ten root sections each). The number of segments with any infection was counted using an Olympus BH2 microscope [40× magnification]. Percentage of roots colonized from sampled roots was calculated as follows [52]

$$\mathrm{root}\;\mathrm{colonization}=\frac{\mathrm{number}\;\mathrm{of}\;\mathrm{colonized}\;\mathrm{root}\;\sec\mathrm{tions}}{\mathrm{total}\;\mathrm{number}\;\mathrm{of}\;\mathrm{root}\;\sec\mathrm{tions}}\times 100\%$$

### 2.8. Leaf Sampling for Chemical Analysis

Oven-dried leaf and root samples were pooled for chemical analysis in the same way as described above for leaf gene expression analysis. The sample tissue was milled to a fine powder using metal beads in the Tissue Lyser II (Qiagen, Hilden, Germany). Samples of c. 2–3 mg of leaf or root material were weighed and put in folded tin foil cups for analysis of C and N using a Flash1112 Elemental Analyzer (Thermo Scientific, Waltham, MA, USA). To determine total amount of phosphorous in plant leaf material, 20 mg of the milled leaves were dissolved in 250 µL of 69% nitric acid and 125 µL of 30% hydrogen peroxide and digested using a closed vessel microwave digester for 100 min at a max temperature of 140 °C. After clear digestion and filtration, the concentration of P was determined using Inductive Coupled Plasma—Atom Emission Spectral analysis ICAP 6500-duo (Thermo Scientific, Waltham, MA, USA) [53].

### 2.9. RNA Extraction and Gene Expression Analysis

From the 18 flash frozen leaf discs per pooled sample, c. six—seven leaf discs were ground with beads in the Qiagen Tissue Lyser II Sample Disrupter for RNA extraction. RNA was extracted and purified following the protocol of RNeasy plant mini kit (Qiagen, Hilden, Germany) and eluted in 50 µL RNase free water. DNA contamination was removed by DNase treatment (DNase I, RNase-free kit, Thermo Scientific, Waltham, Massachusetts, USA). The RNA concentration was measured using a Nanodrop ND-100 spectrophotometer (NanoDrop Technology, Wilmington, DE, USA). The ratio of absorbance at 260 and 280 nm was used to assess RNA purity.

To quantify the relative expression of transcripts for a selected set of marker genes for plant hormonal light and defense signaling pathways (JA, SA, ET, ABA), real-time RT–PCR was performed using the SYBR Green RT-PCR Kit (Bio-Rad Hercules, California, USA). The nucleotide sequences of the primers used and the pathways that these marker genes are involved in are indicated in Supplementary Table S1. Total RNA was isolated from leaves, which had been stored at −80 °C. RNA was reverse transcribed to cDNA using iScript cDNA synthesis kit (Bio-Rad) and stored at −20 °C until further use. Transcripts of marker genes *TD2*, *LoxD* [54], *PAL* [55], *PR6* [56], *GluB* [55], *Le4* [57], *JAZ* [58] and *DELLA* were quantified in a qRT-PCR (CFX96tm Real-Time System, Bio-Rad, Hercules, CA, USA). The thermal cycling was 95 °C for 3 min, followed by 40 cycles of 95 °C for 15 min and 62 °C for 45 s. Expression values were normalized using the housekeeping gene *CAC* [59] which encodes the tomato Clathrin adaptor complexes medium subunit/Endocytic pathway gene. The experiments were independently repeated, and each reaction was performed in duplicate.

### 2.10. Statistical Analysis

All analyses were performed using the R package version 4.0.4. Plant phenotypic data (the response variables height and biomass) were analyzed using linear mixed models with Gaussian distribution (package: lme4) with light, microbial inoculation, and herbivore as independent variables (fixed factors). The whole-plot factor (light) was tested against the whole-plot error term block x light. Block and block x light were entered as random factors in the model. Plant heights measured at different time-points were analyzed separately per time-point. A generalized linear model with binomial distribution was applied to analyze effects of light and herbivory on AMF colonization. Relative expression of genes was calculated using the delta delta Ct method [60] and statistically analyzed using linear mixed models with relative expression as dependent variable and light, microbial inoculation and herbivory as independent variables. Residuals of all linear models were tested for normality and homogeneity of variances using the Shapiro-Wilk and Levene’s tests, respectively. Data for the expression values of four of the genes tested for differences in gene expression were log-transformed to meet these assumptions. Effects of light and microbes on caterpillar RGR were analyzed using generalized linear models with a poisson distribution. Caterpillar survival was analyzed using Cox Proportional Hazard Survival analysis and plotted using a Kaplan-Meier Survival Model (package: survival).

## 3. Results

### 3.1. Effects of Light Quality and Herbivory on Mycorrhizal Colonization and Plant Development

**Figure 1:**
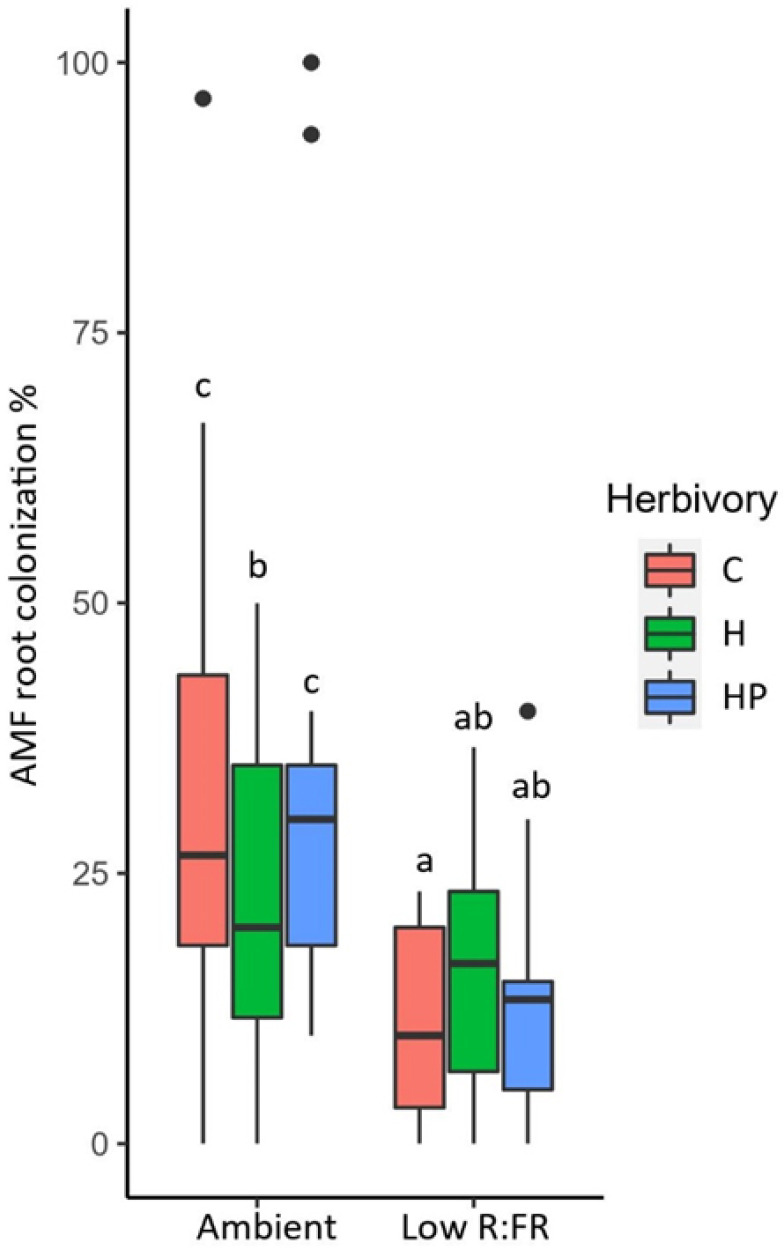
Percentage of tomato roots colonized by AMF under different conditions of light (ambient; low R:FR light) and herbivory. (C = no herbivory; H = herbivory by unparasitized caterpillars; HP = herbivory by parasitized caterpillars). Boxplots that don’t share the same letter are significantly different *p* < 0.05 (Tukey HSD). The median is represented by the thick horizontal line; the box is defined by the 25th and 75th percentiles (lower and upper quartile).

*Solanum lycopersicum* plants grown under continuous low R:FR light for 8 weeks showed significantly lower root colonization by *Rhizophagus irregularis* than plants grown under ambient light. Overall, the average colonization rate was 30% for plants growing under ambient light and 13% for plants in the low R:FR treatment (X^2^(1) = 18.3, *N* = 86, *p* < 0.001). The effect of light quality on AMF colonization depended on parasitization of the herbivore (Figure 1, Table S2). Herbivory by unparasitized caterpillars reduced AMF colonization under ambient light conditions, but not under low R:FR light.

**Figure 2:**
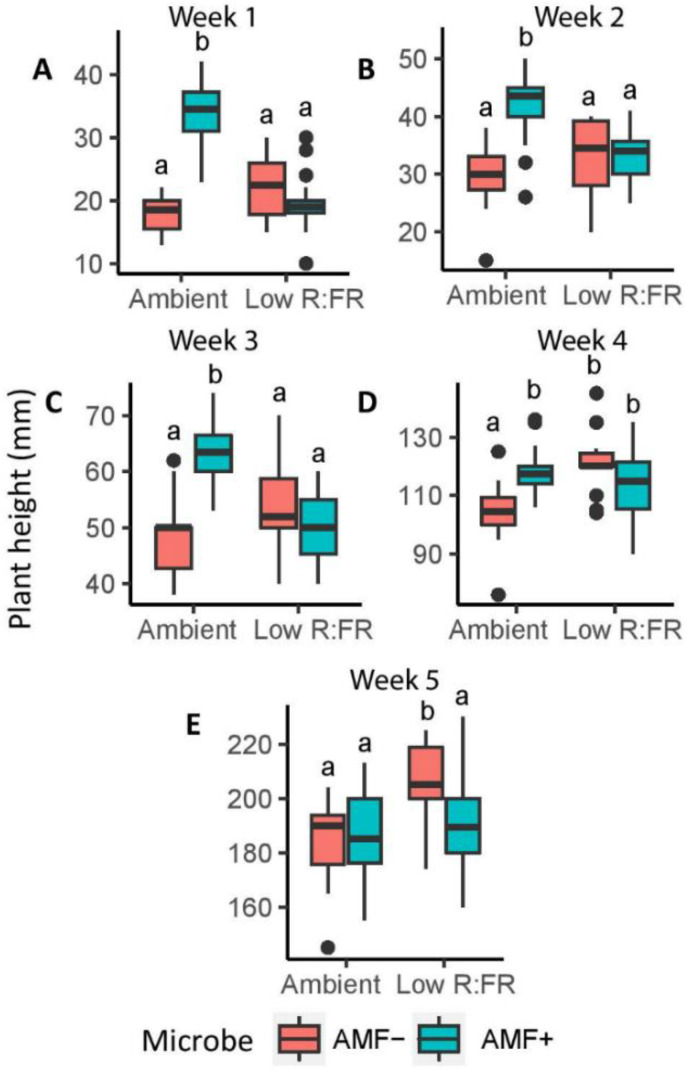
Height of tomato plants during the first five weeks (**A**–**E**) after seedling transplantation grown under two light (Ambient and Low R:FR) and two inoculation treatments (AMF−: no mycorrhizae, AMF+: mycorrhiza). Boxplots that don’t share the same letter are significantly different *p* < 0.05 (Tukey HSD). The median is represented by the thick horizontal line; the box is defined by the 25th and 75th percentiles (lower and upper quartile).

Plant height was significantly higher under the low R:FR treatment than under the ambient light treatment from week one onwards (Table 1), indicating a shade avoidance syndrome response. AMF inoculation significantly increased plant height under ambient light, although the extent of this increase diminished with time. By contrast, AMF did not improve plant height under low R:FR and even reduced plant height by 8% in week five, mitigating the shade avoidance response (F (1,58) = 8.0, *p* = 0.007). Therefore, during all five weeks of measurements there was a strong interaction effect between light quality and the presence of AMF (Figure 2). The herbivory treatment started in the 4th week of measurements and hence effects of this treatment were only analyzed for the last two weeks of measurements. The presence of unparasitized caterpillars significantly decreased plant height across all treatments in week 5 (F (2,194) = 3.3, *p* = 0.039; Table S3).

**Figure 3:**
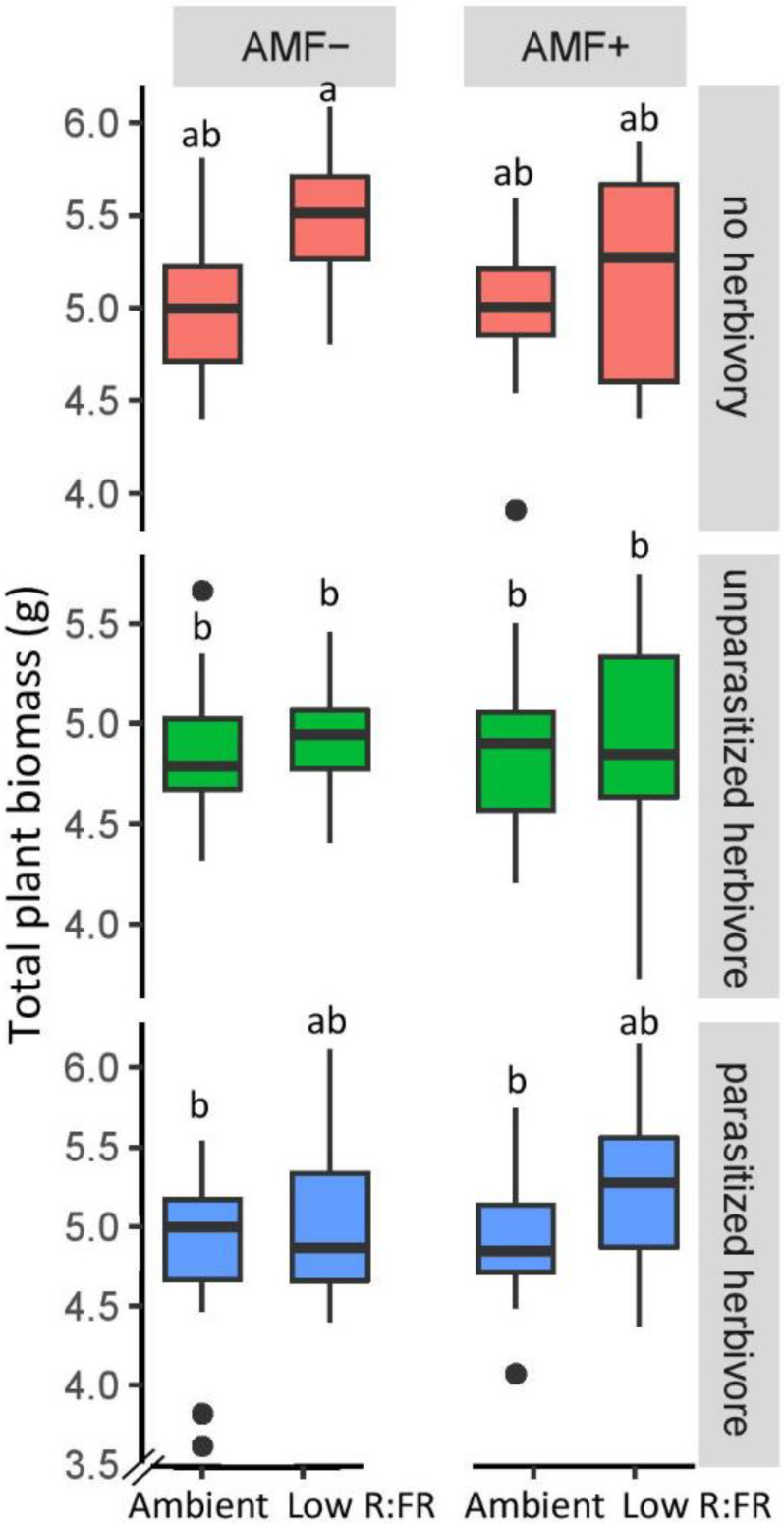
Total dry biomass of tomato plants grown under two light (Ambient, Low R:FR) and two inoculation treatments (AMF−: no mycorrhiza, AMF+: mycorrhiza) and subjected to three herbivory treatments. Boxplots that don’t share the same letter are significantly different *p* < 0.05 (Tukey HSD). The median is represented by the thick horizontal line; the box is defined by the 25th and 75th percentiles (lower and upper quartile).

At harvest, light quality strongly affected total plant biomass (Figure 3), stem biomass (Figure S2a), root biomass (Figure S2b) and root mass fraction (Figure S2c). As observed for stem height, the effect of light quality on stem dry weight depended on inoculation treatment (Table 2). In non-mycorrhizal plants, low R:FR light significantly increased the stem biomass compared to the ambient light treatment, but in mycorrhizal plants it did not (F (1,160) = 12.3, *p* = 0.004). As expected, low R:FR light reduced the root mass fraction (F (1,160) = 16.2, *p* < 0.001; Table 2, Figure S2c), indicating increased resource allocation to shoots under perceived shading, but this effect tended to be smaller in the presence of AMF and herbivory (Table 2).

Light quality and microbe treatment had no effect on leaf biomass, but herbivores significantly reduced leaf biomass (F (2,160) = 5.7, *p* = 0.004; Table 2) and increased root dry biomass (F (2,160) = 6.6, *p* = 0.002), resulting in an increased root weight ratio especially in the presence of mycorrhizae. A negative correlation between total shoot biomass and the percentage of root AMF colonization was observed under ambient light in non-herbivory plants (Figure S3) but not in other treatments. There was no significant three-way interaction between light, inoculation, and herbivory in any of the response variables.

Contrary to expectations, addition of mycorrhizal inoculum did not lead to an increase in leaf phosphorus or nitrogen concentrations. No significant differences in leaf phosphorous concentration were observed across treatments (Table S3; Figure S4). Irrespective of light quality, the addition of mycorrhizal inoculation did lead to a reduction in leaf carbon concentration (F (1,40) = 0.1, *p* = 0.049; Table S3, Figure S5). Plant C:N ratio (root and leaf) was not affected by any treatment.

### 3.2. Effects of Light Quality and AMF Inoculation on the Performance of S. exigua and C. marginiventris

**Figure 4:**
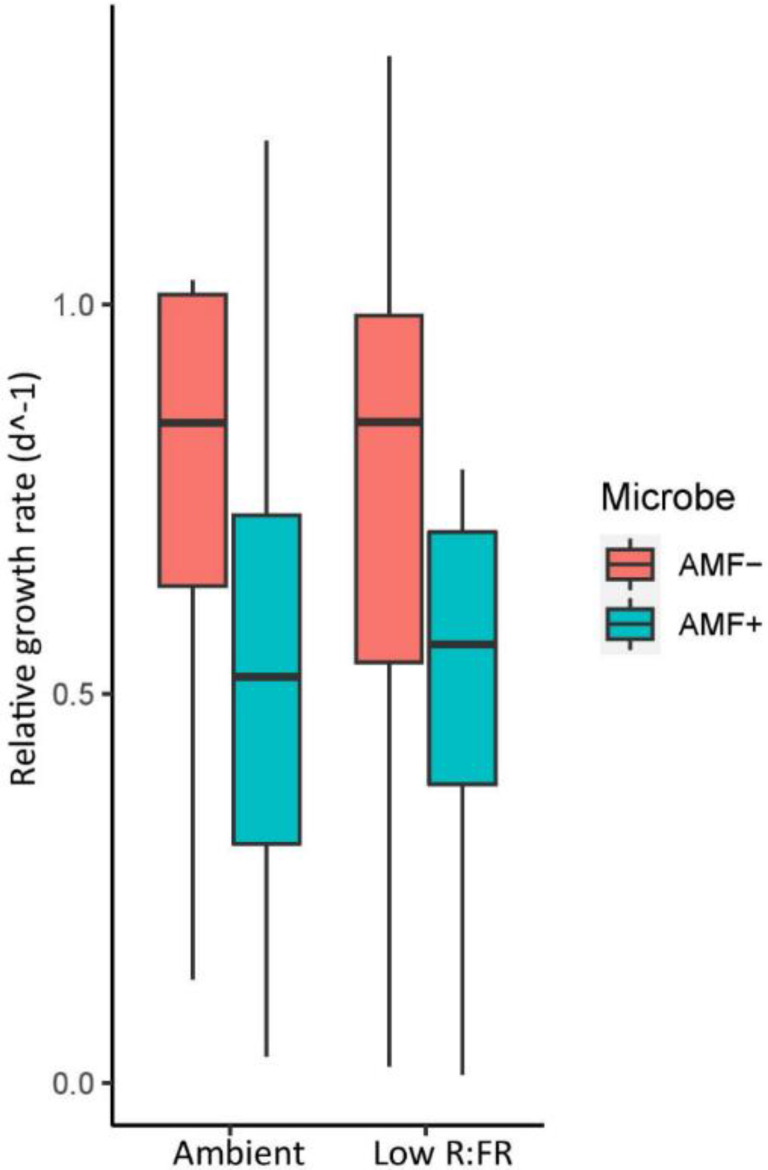
Relative growth rate (RGR) of unparasitized *Spodoptera exigua* feeding on tomato plants grown under two light (Ambient, Low R:FR) and two inoculation treatments (AMF−: no mycorrhiza, AMF+: mycorrhiza). RGR = (lnW[final]) − lnW[initial])/*t*. W = weight, *t* = time in days. The error bar denotes 1SE. No significant differences were observed in RGR across light and inoculation treatments. The median is represented by the thick horizontal line; the box is defined by the 25th and 75th percentiles (lower and upper quartile).

Overall, survival of caterpillars was moderate (72%) for unparasitized caterpillars, and low (38%) for caterpillars that were parasitized prior to parasitoid emergence. AMF inoculation and light treatment did not significantly affect the survival of unparasitized (*p* = 0.45 K-M) or parasitized caterpillars (*p* = 0.44 K-M) (Table S4, Figure S6). However, the RGR of surviving unparasitized caterpillars tended to be lower on mycorrhizal compared to non-mycorrhizal plants (F (1,32) = 3.87, *p* = 0.057; Table S5, Figure 4). Furthermore, the proportion of parasitized caterpillars that produced a parasitoid cocoon was independent of light and inoculation treatments. Treatment effects on herbivory were not reflected in cocoon weight, adult parasitoid weight, or sex.

### 3.3. Effects of Light Quality and AMF Inoculation on Plant Defense Signaling in Response to Herbivory

**Figure 5:**
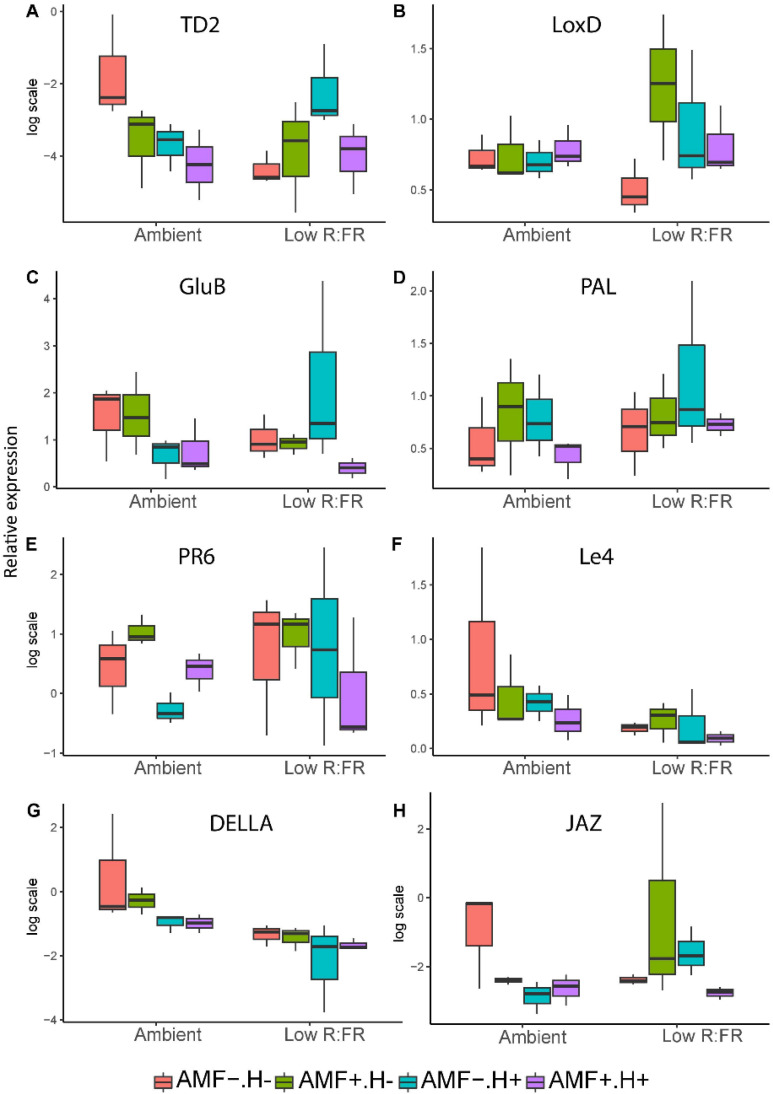
Relative expression of eight genes (**A**–**H**) related to light and defense signaling in tomato plants grown under two light (Ambient, Low R:FR) and two inoculation treatments (AMF−: no mycorrhiza, AMF+: mycorrhiza) in the absence (H−) or presence (H+) of herbivory by unparasitized *Spodoptera exigua* caterpillars. Values are delta-delta-Ct values normalized to the expression of the housekeeping gene CAC. The median is represented by the thick horizontal line; the box is defined by the 25th and 75th percentiles (lower and upper quartile).

The transcript levels of the targeted genes were analyzed in systemic leaves of plants whose local leaves had (or had not) been exposed to 14 days of herbivory by unparasitized caterpillars. Contrary to our expectation, genes involved in jasmonic acid (JA) and ethylene (ET)-signaled defense responses were not strongly upregulated in response to herbivory under ambient light. These genes also did not show a weaker response to herbivory under low R:FR light than under ambient light conditions. On the contrary, in response to herbivory, non-mycorrhizal plants showed induction of TD2 under low R:FR light (F (1,4) = 9.31, *p* = 0.038), but not under ambient light conditions (non-significant reduction, F (1,4) = 4.52, *p* = 0.101), resulting in a significant light x herbivory interaction (F (1,16) = 6.8, *p* = 0.019; Table S6, Figure 5). Interestingly, the induction of TD2 under low R:FR light was not observed in mycorrhizal plants (F (1,4) = 0.01, *p* = 0.924), indicating that AMF mitigated the induction of this defense gene by the herbivore. The pattern for *LOXD* under low R:FR light conditions was qualitatively similar to that of TD2, but here the effects were not significant. As expected, the transcript levels of the *DELLA* gene, that suppresses the shade avoidance syndrome under ambient light, were higher under ambient light conditions than under low R:FR light (F (1,16) = 12.5, *p* = 0.003). Interestingly, the presence of herbivory repressed the expression of the *DELLA* gene across all treatments (F (1,16) = 5.4, *p* = 0.034). but this was not accompanied by enhanced expression of the investigated *JAZ* gene, which showed an overall slightly reduced expression under herbivory (F (1,16) = 5.3, *p* = 0.036), depending on light and herbivory treatment, AMF further reduced expression of *JAZ* gene (F (1,16) = 7.4, *p* = 0.015). This indicates that the expected light-mediated growth-defense trade-off either did not occur at the investigated timepoint, or that it might be regulated by genes that were not tested in this experiment. Expression of the abscisic acid (ABA)-inducible gene *Le4* showed a different pattern, having an overall strongly reduced expression under low R:FR light (F (1,16) = 8.1, *p* = 0.012; Table S6, Figure 5). The genes involved in SA-signaling (*PAL*, *PR6*) did not respond to any of the treatments.

## 4. Discussion

We tested the interactive effects of light quality and inoculation with arbuscular mycorrhizal fungi on growth and defense of tomato plants. The three main findings were: (1) As expected, low R:FR light induced the shade avoidance syndrome, reduced *R. irregularis* colonization of tomato plants and increased plant total biomass production but unexpectedly, AMF counteracted the plant’s SAS response under low R:FR light; (2) AMF did not significantly affect caterpillar or parasitoid performance; (3) AMF did not induce or prime expression of defense genes. Contrary to expectations, induction of defense gene expression in response to herbivory was only observed for non-mycorrhizal plants under low R:FR light, but under these light conditions defense induction was suppressed by AMF. Overall, these results do not support the hypothesis that AMF mitigates trade-offs between growth and defense under light conditions that induce plant shade avoidance responses.

### 4.1. Plant Growth Is Determined by Interactive Effects of Light Quality and Mycorrhizal Colonization

Our results show that plant height was affected by an interplay between light quality effects and AMF inoculation. Non-mycorrhizal plants increased their height in response to low R:FR light, the classical shade avoidance response. However, whereas AMF inoculation enhanced plant height under ambient light conditions, it reduced plant height in five-week-old plants grown under low R:FR light, thus mitigating the shade avoidance response. Opposing effects of AMF and low R:FR light on plant height have been reported before [61], however without any interaction between light and AMF. Our study therefore reveals more complex, interactive effects of light and AMF, indicating that the beneficial or detrimental effects of AMF on growth are contingent upon light conditions.

The effects of AMF on plant height under low R:FR light could only be partly explained by the observed reduced colonization rate since plant height was not simply less stimulated, but actually reduced by AMF under low R:FR light. Reductions in AMF colonization under low R:FR have been documented in several plant species, including tomato, the grass *Festuca rubra* [61] and the legume *Lotus japonicus* [29]. These reductions have been attributed to the reduction in jasmonic acid and strigolactone production under low R:FR conditions that are important in plant-AMF signaling and stimulate fungal development [29]. Other studies have attributed the low colonization in shaded plants to carbon limitation in the plant itself [32,62]. We expected that plants experiencing leaf herbivory would even more strongly reduce AMF colonization under shade since herbivory imposes another carbon demand under these carbon-limiting conditions [63,64]. However, this effect was only evident in ambient light and not under low R:FR light conditions.

The interactive effects of light and AMF on plant height were also reflected in plant stem biomass at final harvest. Plant stem biomass of herbivore-free plants was enhanced under low R:FR light in non-mycorrhizal plants but not in mycorrhizal plants. The increased stem biomass production of non-mycorrhizal plants under R:FR indicates that the shade avoidance response was not just based on cell elongation but also involved increased shoot biomass production. In contrast, total plant biomass of herbivore-free plants was overall enhanced under low R:FR light, independent of AMF. Positive effects of low R:FR ratio on plant dry weight have been documented in soybean [65] and other plant species [66,67], but is not a universal observation [61].

Although in our experiments AMF did not affect total plant biomass, a negative correlation was observed between shoot biomass and AMF colonization under ambient light conditions. Such negative associations between mycorrhizal colonization and plant growth have been previously observed, both for shoot biomass production [68] and for shoot regrowth after defoliation [6] and have been explained by the carbon drain for the maintenance of AM symbiosis [69,70]. Possibly, the absence of such a relationship under low R:FR light can be explained by the overall low rates of colonization under these conditions, confined to a range (0–25%, Figure S3B) where decreases in plant weight with percent AMF root colonization are not yet observed (Figure S3A). In our experiment, herbivory reduced overall leaf and stem weight but enhanced root weight, resulting in an increase in root mass fraction, in agreement with the idea of ‘herbivore-induced resource sequestration’, storing biomass away from the site of herbivore attack [71].

Mycorrhizal inoculation had minor effects on leaf primary metabolite concentrations, which can be important determinants of insect herbivory. AMF significantly decreased leaf carbon concentrations, irrespective of light and herbivory conditions. Possibly, the increased stem biomass production in response to AMF created a carbon sink that led to a lower leaf carbon concentration, but this remains to be tested. Furthermore, AMF-inoculated, and non-inoculated plants had similar leaf phosphorus and nitrogen concentrations. This contrasts with many studies showing increased leaf P and N concentration in mycorrhizal plants [72,73]. This does not necessarily mean that enhanced P and N uptake through the mycorrhizal pathway (MP) did not occur, but either indicates that this was accompanied by a strong repression of nutrient uptake through the plant’s own roots system (direct pathway, DP) [74], or that nutrients were more efficiently metabolized.

### 4.2. Insect Performance Is Not Strongly Affected by Light Quality and AMF Inoculation

The survival of caterpillars and the number of wasp cocoons produced by parasitized caterpillars were not significantly affected by light or inoculation treatments, although AMF tended to reduce the weight gain of unparasitized caterpillars. The absence of significant AMF-induced resistance of plants in our experiment adds to a growing number of studies where beneficial microbes fail to benefit plant defense [24,75]. These results suggest that AMF-induced resistance to generalist chewing herbivores, that is observed in several systems [9], is not a universal pattern, and that AMF benefits to plants in terms of damage reduction are not unequivocal. A recent review [12] revealed no consistent patterns in the effect of AM fungi on plant-herbivore interactions, whereas an earlier meta-analysis [26] had reported that leaf chewing herbivores even performed better on mycorrhizal plants but without increasing damage, presumably due to the lower survival of these herbivores on mycorrhizal plants. Opposite to those findings, we report a slightly reduced growth rate without significant effects on survival, highlighting the context dependency of such interactions.

The absence of AMF effects on parasitoid cocoon production or parasitoid weight at eclosion likely indicates that in our experiment, *R*. *irregularis* did not influence parasitoid development. Studies exploring the effects of AMF on the rate of parasitism [46] or on host preference by parasitoids [76] have highlighted that interactions are species specific. Studies of AMF effects on parasitoid development or performance are scarce. Faster development and increased weight of the aphid parasitoid *Aphidius rhopalosiphi* was reported on plants inoculated with the AMF *Funneliformis mosseae* [5]. In our study we did not observe such effects, most likely due to low survival of the host caterpillars. This indicates a ‘non-reproductive host-killing’ behavior of the parasitoid as observed in various parasitoid taxa on holometabolous (Lepidoptera, Coleoptera, Diptera) and hemimetabolous host taxa (Hemiptera) [77]. This phenomenon can be ascribed to e.g., environmental effects such as temperature, immune defense costs, aborted parasitism, or ecological and evolutionary incompatibility between host and parasitoid [77].

### 4.3. AMF-Induced Resistance and AMF-Induced Changes in Gene Expression Are Context-Dependent

In agreement with the weak effects of AMF on caterpillar performance and the absence of increased susceptibility of plants under low R:FR light, we did not observe a strong induction of the tested set of JA and ET defense genes upon herbivory attack by AMF, nor did we observe a depression of defense gene expression under low R:FR light due to SAS. Such a depression was expected in compliance with the growth-defense trade off in SAS plants [78,79,80,81]. Contrary to expectation, the JA and ET responsive biosynthesis and defense genes tested tended to be induced by insect feeding, but only in the low R:FR light treatment.

In *Arabidopsis thaliana*, low R:FR light results in inactivation of phytochrome B, leading to activation of Phytochrome Interaction Factors (PIFs) that repress *DELLA* proteins. Lower expression of *DELLA* proteins subsequently activates SAS and upregulates *JAZ* repressors of JA-signaled defense [79], resulting in lower expression of defense genes. The higher herbivore-induced expression of TD2 under low R:FR than under ambient light that we observed in tomato is therefore unexpected. We did observe reduced expression of the *DELLA* protein under low R:FR light, but this was not accompanied by a corresponding induction of the herbivore-responsive *JAZ* protein that we selected. We can only speculate why we observed higher expression of *TD2* under low R:FR light conditions. Perhaps in tomato the regulation of this defense gene is controlled by the interplay between different light signaling components (other than phytochrome interacting factor (PIFs)), and *DELLA* proteins, and thus does not completely impair the *JAZ*-dependent defense.

Furthermore, most experiments investigating mechanisms underlying interactions between light signaling and defense have been performed using young plants shortly after initiation of light and herbivory treatments. Perhaps after a prolonged period of supplemented far-red light, the growth-defense trade off response is attenuated, resulting in the absence of an effect of the light quality treatments on herbivore performance at least via these signaling defense pathways. Similarly, the effect of low R:FR on plant phenotypic traits remained significant but decreased over time, indicating that low R:FR light-induced growth-defense trade-offs may also diminish with time.

The results of the gene expression analyses are not in line with the expected outcome of a mycorrhiza-mediated increase in plant defense against herbivory [14,82]. AMF inoculation usually leads to enhanced activation of defense genes such as β-1,3-glucanases (*GluB*), chitinases, phenylalanine ammonia-lyase (*PAL*) and lipoxygenase (*LoxD*) in tomato leaves upon biotic stress [10,14,83]. By contrast, in this study, AMF did not affect expression of defense genes under ambient light, and even repressed their induction under low R:FR light. There may be several reasons why we did not observe priming by AMF or an overall induction in defense response genes upon herbivory. First, leaves were sampled on the 14th day after initiation of herbivory, when resistance gene responses could already have been attenuated. Second, plants were relatively old and defense responses may decrease with ontogenetic stage of the plants [84].

## 5. Conclusions

We conclude that light quality affects the impact of symbiosis with mycorrhizal fungi on plant growth and plant–herbivore interactions. Low R:FR strongly modulated AMF root colonization and, colonization rate in turn affected plant traits by influencing plant growth and biomass. Mycorrhizal inoculation did not significantly affect caterpillar weight gain over time and did not affect parasitoid emergence and development. There was no evidence for priming of JA defenses by AMF, and AMF even mitigated herbivore-induced defense gene activation under low R:FR light. It should be noted that a potential caveat of our study is that we only used a single widely studied species of AMF, and that studies of a broader set of AMF species is necessary to generalize conclusions about AMF effects on plant-enemy interactions [85]. Several controlled growth chamber experiments show effectiveness of applying mycorrhizal products in stimulating plant and improving resistance; however, a growing number of studies have also highlighted their unpredictability [19,86]. As sustainable agriculture remains priority, our results contribute to understanding the ecology of plant-beneficial microbe-pest interactions and predicting their potential application.

## Figures and Tables

**Table 1:** General linear mixed models of the effects of light and microbial inoculation on no-herbivore plant height during early growth.

	Week 1			Week 2		Week 3		Week 4		Week 5	
Treatments	n,ddf	F	*p*	F	*p*	F	*p*	F	*p*	F	*p*
L: Light	1,5	23.9	**0.005**	3.0	0.146	5.1	0.074	6.1	0.057	9.3	**0.028**
M: Microbe	1,58	38.5	**<0.001**	24.5	**<0.001**	14.1	**<0.001**	2.4	0.131	3.0	**0.091**
L × M	1,58	71.6	**<0.001**	18.7	**<0.001**	32.8	**<0.001**	21.5	**<0.001**	8.0	**0.007**

All values in bold indicate differences significant at *p* < 0.05.

**Table 2:** General linear mixed models of the effects of light, microbial inoculation, and herbivory on dry weight production of different plants parts and on root mass fraction (RMF) at harvest.

		Total		Root		Stem		Leaf		Inflorescence		RMF	
Treatments	n,ddf	F	*p*	F	*p*	F	*p*	F	*p*	F	*p*	F	*p*
L: Light	1,4	8.1	**0.046**	2.8	0.093	53.7	**<0.001**	0.1	0.937	1.0	0.346	16.2	**<0.001**
M: Microbe	1,160	0.0	0.826	1.9	0.169	4.5	**0.034**	0.6	0.418	18.1	**<0.001**	2.2	0.131
H: Herbivore	2,160	5.6	**0.004**	6.6	**0.002**	5.7	**0.004**	13.3	**<0.001**	1.1	0.330	20.2	**<0.001**
L × M	1,160	0.2	0.647	2.1	0.145	12.3	**0.004**	3.3	0.069	3.7	0.053	3.8	0.051
L × H	2,160	1.3	0.271	0.1	0.916	0.6	0.505	1.6	0.204	0.4	0.666	0.1	0.910
M × H	2,160	1.7	0.183	2.5	0.084	3.5	**0.030**	0.2	0.752	10.1	**<0.001**	3.2	**0.041**
L × M × H	2,160	1.4	0.238	1.1	0.306	1.4	0.230	0.1	0.819	1.4	0.241	0.3	0.714

All values in bold indicate differences significant at *p* < 0.05.

## Data Availability

The data that supports the finding of this study will be made available open through the Dryad digital repository upon acceptance.

## Supplementary Materials

The following supporting information can be downloaded at: https://www.mdpi.com/article/10.3390/plants11070861/s1, Figure S1: Measurements of light spectrum in the glasshouse; Figure S2: Dry biomass of tomato plants after eight weeks of growth; Figure S3: Regression of shoot dry weight on % root colonization by AMF; Figure S4: Leaf phosphorus concentration; Figure S5: Leaf carbon concentration; Figure S6: Kaplan-Meier survival curves; Table S1: Primer sequences used in the gene expression analysis; Table S2: Effect of light on AMF colonization; Table S3: Effect of light, AMF and herbivory on plant growth; Table S4: Effect of light, AMF and herbivory on leaf phosphorus and carbon concentrations; Table S5: Cox Proportional Hazard survival analysis; Table S6: Relative growth rate of S. exigua; Table S7: Effect of light, AMF and herbivory on targeted gene expression.
