# Supplementary material for: Effects of Light Quality on Colonization of Tomato Roots by AMF and Implications for Growth and Defense
Source: Plants (Basel). 2022 Mar 24;11(7):861. doi: 10.3390/plants11070861 (PMC9002964; doi:10.3390/plants11070861)
Supplement: Supplementary file 1 [file plants-11-00861-s001.zip › plants-1617239-supplementary.pdf]

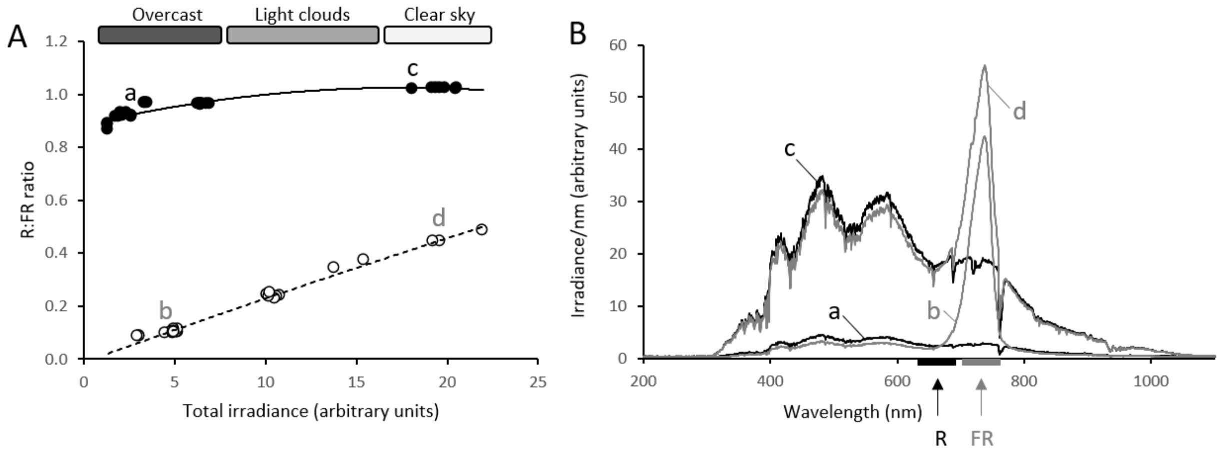

Figure S1. Measurements of light spectrum in the glasshouse. (**A**): Red-to-far-red ratio of light intercepted at plant level for plants that received ambient light (black dots, solid line) or that were supplemented with far-red LED light (open dots, dotted line) in a greenhouse under different outside weather conditions during the day. Data were collected at random timepoints between 10:00 and 14:00 on a day with variable cloudiness (overcast, light clouds, clear sky) and were plotted against total irradiance. (**B**): Light spectra for datapoints a-d indicated in figure (**A**) (a: ambient light, cloudy; b: FR-supplemented light, cloudy; c: ambient light, full sun; d: FR supplemented light, full sun). R:FR ratios were calculated as the ratio of irradiance measured between 630 and 660 nm (red) to the irradiance measured between 700 and 760 nm (far-red). Spectra were measured using an AvaSpec Mini2048 photospectrometer (Avantes, Apeldoorn NL) and analyzed using Avasoft 8.9 software. Values are corrected for dark spectra and expressed as ADC counts (**B**) or ACD counts * 10^6 (**A**).

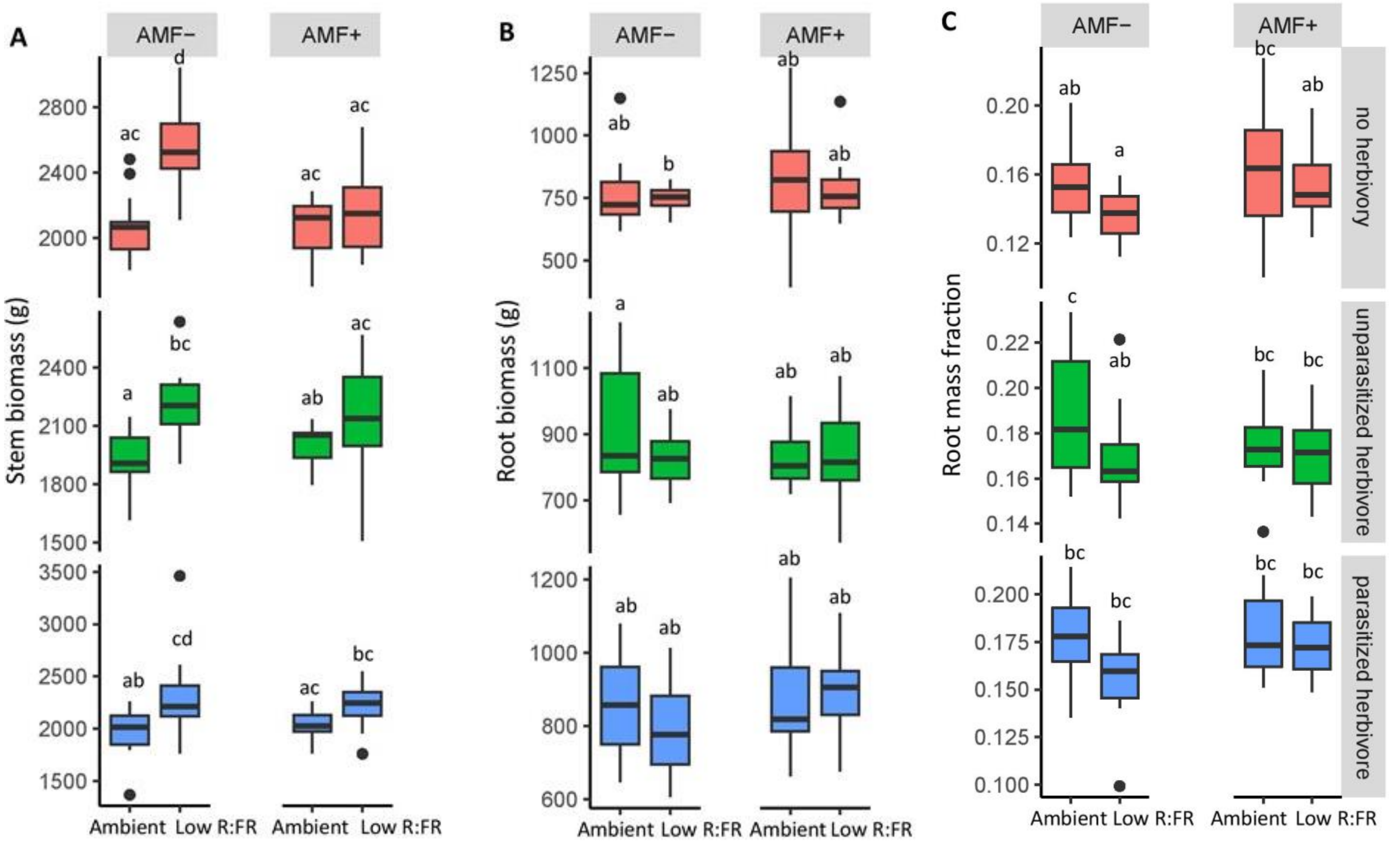

Figure S2. Dry biomass of tomato plants after eight weeks of growth. Stem (**A**), root (**B**) and root mass fraction (**C**) of plants grown under two light (Ambient, Low R:FR) and two inoculation treatments (AMF-: no mycorrhiza, AMF+: mycorrhiza) and subjected to three herbivory treatments (red = no herbivory; green = herbivory by unparasitized caterpillars; blue = herbivory by parasitized caterpillars). Boxplots (within a panel) that don't share the same letter are significantly different $p<0.05$ (Tukey HSD). The median is represented by the thick horizontal line; the box is defined by the 25th and 75th percentiles (lower and upper quartile).

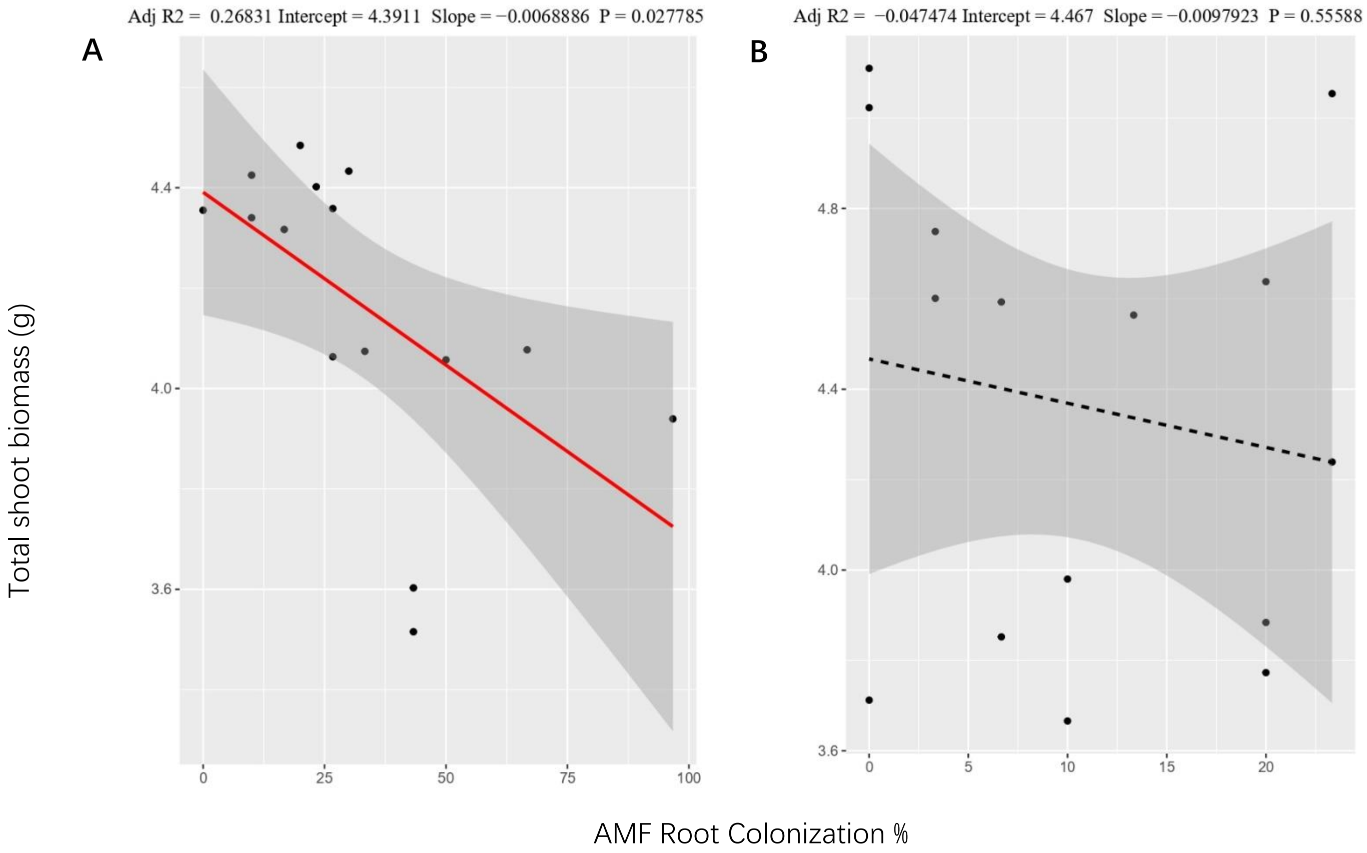

Figure S3. Regression of shoot dry weight on % root colonization by AMF for tomato plants grown under (**A**) ambient light and (**B**) low R:FR light.

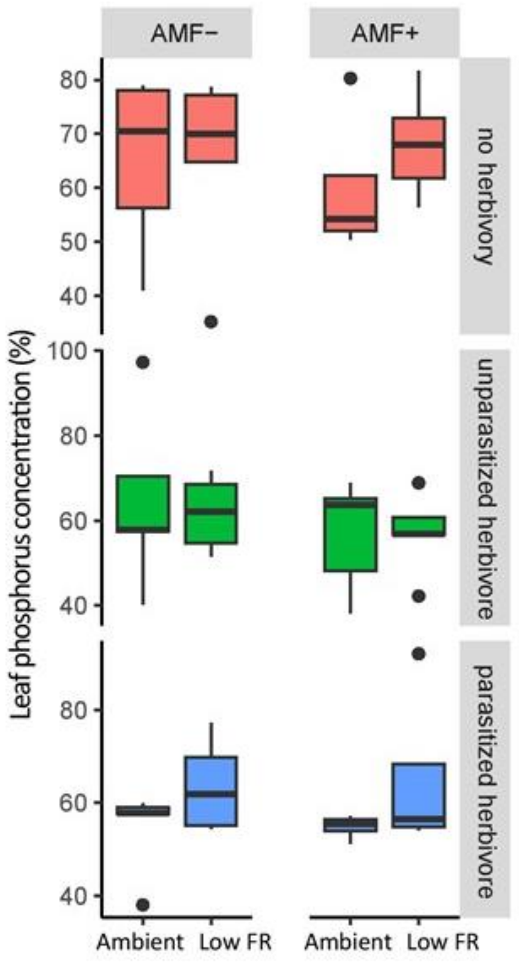

Figure S4. Leaf phosphorus concentration of tomato plants grown under two light (Ambient, Low R:FR) and two inoculation treatments (AMF-: no mycorrhiza, AMF+: mycorrhiza) and subjected to three herbivory treatments. No significant differences were observed in phosphorus concentration per cent across light inoculation and herbivory treatments. The median is represented by the thick horizontal line; the box is defined by the 25th and 75th percentiles (lower and upper quartile).

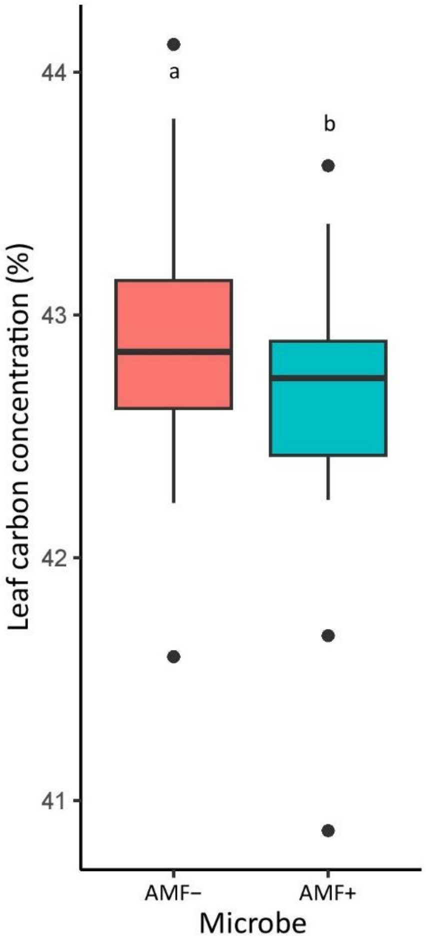

Figure S5. Leaf carbon concentration of tomato plants grown under two inoculation treatments (AMF-: no mycorrhiza, AMF+: mycorrhiza). Since light and herbivory treatments did not affect this trait, data have been summarized across these treatments. Boxplots that don't share the same letter are significantly different $p<0.05$ (Tukey HSD). The median is represented by the thick horizontal line; the box is defined by the 25th and 75th percentiles (lower and upper quartile).

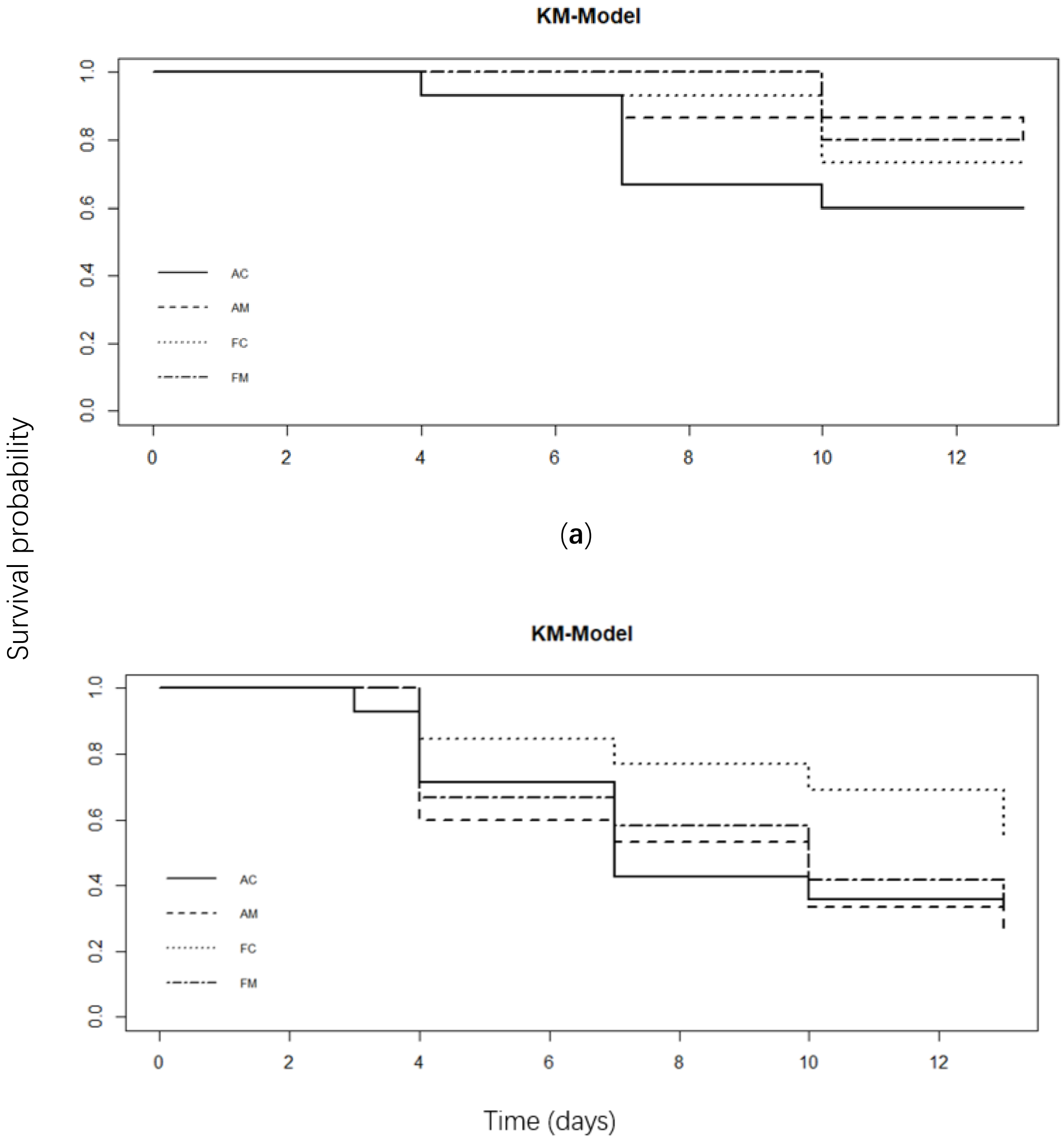

(**b**)

Figure S6. Kaplan-Meier survival curves of (**a**): unparasitized and (**b**): parasitized caterpillars feeding on tomato plants grown under two light and two inoculation treatments (AC: ambient light, no mycorrhiza; AM: ambient light, mycorrhiza; FC: low R:FR light, no mycorrhiza; FM: low R:FR light, mycorrhiza).

Table S1. Primer sequences used in the gene expression analysis. The genes monitored are used as markers for the pathways indicated. Jasmonate (JA), salicylic acid (SA), abscisic acid (ABA), and ethylene (ET).

ID	Target Gene	Related to	Pathway Primer (5' – 3')
Solyc08g006960.2.1	CAC	Housekeeping	CCTCCGTTGTGATGTAACTGG<br>ATTGGTGGAAAGTAACATCATC
AAA68097.1	TD2	Wound/<br>JA inducible	TGCCGTTAAAAATGTCACCA<br>ACTGGCGATGCCAAAATATC
NM001320292	LoxD	JA biosynthesis	GACTGGTCCAAGTTCACGATCC<br>ATGTGCTGCCAATATAAATGGTTCC
NM001247876	GluB	ET inducible	CCATCACAGGGTTCATTTAGG<br>CCATCCACTCTCTGACACAACT
M83314	PAL	SA biosynthesis	CGTTATGCTCTCCGAACATC<br>GAAGTTGCCACCATGTAAGG
NM001247385.2	PR6	SA inducible	GGATCGGACAACGTCCTTAC<br>GCAACATCAAAAGGGAAATAAT
X51904	Le4	ABA inducible	ACTCAAGGCATGGGTACTGG<br>CCTTCTTTCTCCTCCCACCT
Solyc11g011260	Della (procera)	Negative gibberellin<br>regulator	CACAAGAAACTGGGGTTCGT<br>CAGATTGTGAAACCGCAAGA
Solyc12g009220.1.1	JAZ	Repressor of JA<br>signaling pathway	CGTCCGTTGAAACAAATCCT<br>GGGGTTCTGTTTGTTGGCTA

Table S2. Generalized linear model of the effects of light (ambient vs. low R:FR light) and herbivory (none, unparasitized caterpillars, parasitized caterpillars) on the colonization of tomato roots by *R.irregularis*.

Treatment	df	Chisq	p
L: Light	1	18.3	<**0.001**
H: Herbivory	2	5.8	0.053
L x H	2	18.9	<**0.001**

Table S3 Linear mixed models of the effects of light (ambient vs. low R:FR light), microbial inoculation (with or without AMF) and herbivory (none, unparasitized caterpillars, parasitized caterpillars) on plant height during two weeks of growth.

		Week 4		Week 5	
Treatment	ndf,ddf	F	p	F	p
L: Light	1,4	8.3	**0.031**	15.8	**0.011**
M: Microbe	1, 194	16.1	<**0.001**	0.0	0.940
H: Herbivory	2, 194	0.8	0.445	3.3	**0.039**
L x M	1, 194	46.3	<**0.001**	17.4	<**0.001**
L x H	2, 194	0.1	0.910	1.2	0.279
M x H	2, 194	0.7	0.477	2.4	0.092
L x M x H	2, 194	0.4	0.646	0.5	0.588

Table S4. Linear models of the effects of light (ambient vs. low R:FR light), microbial inoculation (with or without AMF) and herbivory (none, unparasitized caterpillars, parasitized caterpillars) on leaf phosphorus and carbon concentrations at harvest.

		Phosphorus		Carbon	
Treatment	n,ddf	F	p	F	p
L: Light	1,4	2.1	0.316	0.0	0.836
M: Microbe	1,40	0.1	0.505	4.1	**0.049**
H: Herbivory	2,40	0.7	0.004	1.3	0.267
L x M	1,40	0.1	0.732	0.0	0.852
L x H	2,40	0.8	0.143	0.4	0.651
M x H	2,40	0.5	0.085	0.2	0.814
L x M x H	2,40	0.1	0.890	2.5	0.092

Table S5. Survival analysis based on Cox Proportional Hazard models for the effects of light (ambient vs. low R:FR light) and microbial inoculation (with or without AMF) of host plants on the survival of non-parasitized and parasitized caterpillars of *Spodoptera exigua*.

		Unparasitized		Parasitized	
Treatment	df	Chi-sq	p	Chi-sq	p
L: Light	1	0.5	0.451	1.2	0.256
M: Microbe	1	1.6	0.204	0.9	0.328
L x M	1	0.2	0.613	0.4	0.506

Table S6. Linear models of the effects of light (ambient vs. low R:FR light) and microbial inoculation (with or without AMF) of host plants on the relative growth rate (RGR) of non-parasitized and parasitized caterpillars of *Spodoptera exigua*.

		Unparasitized		Parasitized	
Treatment	ndf,ddf	F	p	F	p
L: Light	1,4	0.01	0.941	1.47	0.291
M: Microbe	1,32	3.87	0.057	2.42	0.127
L x M	1,32	0.02	0.895	0.51	0.479

Table S7. Linear models of the effects of light (ambient vs. low R:FR light), microbial inoculation (with or without AMF) and herbivory (none, unparasitized caterpillars, parasitized caterpillars) on the expression of genes involved in light- and defense signaling in tomato leaves.

		TD2		LOXD		GluB		PAL	
Treatment	n,ddf	F	p	F	p	F	p	F	p
L: Light	1,16	0.4	0.510	1.1	0.314	0.0	0.980	1.2	0.302
M: Microbe	1,16	4.2	0.057	2.1	0.162	1.4	0.252	0.2	0.613
H: Herbivory	1,16	0.1	0.763	0.0	0.972	0.5	0.504	0.1	0.743
L x M	1,16	0.3	0.542	1.1	0.315	1.9	0.181	0.1	0.783
L x H	1,16	6.8	**0.019**	0.0	0.993	2.3	0.147	0.6	0.425
M x H	1,16	0.2	0.594	2.6	0.125	1.2	0.284	2.9	0.108
L x M x H	1,16	3.9	0.064	3.6	0.077	1.4	0.252	0.0	0.966

		PR6		Le4		DELLA		JAZ	
Treatment	n,ddf	F	p	F	p	F	p	F	p
L: Light	1,16	0.3	0.566	8.1	**0.012**	12.5	**0.003**	1.2	0.282
M: Microbe	1,16	0.3	0.584	0.8	0.369	0.1	0.809	0.9	0.369
H: Herbivory	1,16	1.4	0.148	2.6	0.125	5.4	**0.034**	5.3	**0.036**
L x M	1,16	2.3	0.256	0.2	0.656	0.8	0.395	0.1	0.820
L x H	1,16	0.1	0.739	0.1	0.819	0.6	0.463	1.9	0.185
M x H	1,16	0.5	0.504	0.3	0.603	0.9	0.361	0.3	0.580
L x M x H	1,16	0.6	0.462	0.0	0.877	0.0	0.956	7.4	**0.015**
